# PPARγ/SOD2 Protects Against Mitochondrial ROS-Dependent Apoptosis via Inhibiting ATG4D-Mediated Mitophagy to Promote Pancreatic Cancer Proliferation

**DOI:** 10.3389/fcell.2021.745554

**Published:** 2022-02-02

**Authors:** Shuang Nie, Zhao Shi, Mengyue Shi, Hongzhen Li, Xuetian Qian, Chunyan Peng, Xiwei Ding, Shu Zhang, Ying Lv, Lei Wang, Bo Kong, Xiaoping Zou, Shanshan Shen

**Affiliations:** ^1^ Department of Gastroenterology, the Affiliated Drum Tower Hospital of Nanjing University Medical School, Nanjing, China; ^2^ Nanjing University Institute of Pancreatology, Nanjing, China; ^3^ Department of Gastroenterology, Nanjing Drum Tower Hospital, Clinical College of Nanjing Medical University, Nanjing, China; ^4^ Department of Surgery, Ulm University Hospital, Ulm University, Ulm, Germany

**Keywords:** PPARγ, SOD2, ATG4D, mitophagy, pancreatic ductal adenocarcinoma

## Abstract

Pancreatic ductal adenocarcinoma (PDAC) is an extremely aggressive disease with poor prognosis. Our previous study found that peroxisome proliferator activated receptor gamma (PPARγ) was capable of enhancing glycolysis in PDAC cells. However, whether PPARγ could promote PDAC progression remains unclear. In our present study, PPARγ was positively associated with tumor size and poor prognosis in PDAC patients. Functional assays demonstrated that PPARγ could promote the proliferation of pancreatic cancer cells *in vitro* and *in vivo*. Additionally, flow cytometry results showed that PPARγ decreased mitochondrial reactive oxygen species (mitochondrial ROS) production, stabilized mitochondrial membrane potential (MMP) and inhibited cell apoptosis *via* up-regulating superoxide dismutase 2 (SOD2), followed by the inhibition of ATG4D-mediated mitophagy. Meanwhile, the activation of PPARγ might reduce pancreatic cancer cell stemness to improve PDAC chemosensitivity *via* down-regulating ATG4D. Thus, these results revealed that PPARγ/SOD2 might protect against mitochondrial ROS-dependent apoptosis *via* inhibiting ATG4D-mediated mitophagy to promote pancreatic cancer proliferation, further improving PDAC chemosensitivity.

## Introduction

Pancreatic ductal adenocarcinoma (PDAC) is a lethal cancer with a high mortality rate, the 5-year survival rate of which is only 10% (Siegel et al., 2021). It is probably due to the fact that PDAC is a complex and heterogenic disease with extensive variations in genetic, clinical and histological profiles. It is of great importance to elucidate the molecular mechanisms and to identify new therapeutic strategies for PDAC. We previously generated several novel genetically engineered mouse models (GEMMs) of PDAC entities (Kong et al., 2015; Cheng et al., 2016; Kong et al., 2016; Kong et al., 2018). In a PDAC subtype with poor prognosis characterized by elevated level of ALDH1A3 (aldehyde dehydrogenase family 1, subfamily A3) (Kong et al., 2016; Nie et al., 2020), PPARγ (peroxisome proliferator activated receptor γ) was significantly upregulated, leading to activation of the PI3K/AKT/mTOR signaling pathway and accelerated glycolysis (Nie et al., 2020). However, the specific effects of PPARγ on PDAC malignant behaviors remain unclarified in our previous studies (Nie et al., 2020). PPARγ, as a nuclear receptor transcription factor, regulates mitochondrial function and participates in cancer cell metabolism, oxidative redox and biosynthesis (Calvier et al., 2017; Tseng et al., 2019; Zou et al., 2019). The functions of PPARγ in cancer development is ambiguous, and its roles in PDAC carcinogenesis and progression remain unclear (Nakajima et al., 2008; Reka et al., 2010; Lv et al., 2019; Zou et al., 2019). Thus, it is necessary to further explore the role of PPARγ in PDAC, so as to provide a theoretical basis for therapeutic strategies.

As a lysosomal-dependent degradation pathway, mitophagy selectively targets mitochondria for elimination and renewal (Kubli and Gustafsson, 2012). The regulation of mitochondrial function largely depends on mitophagy (Lesmana et al., 2016; Humpton et al., 2019). On one hand, mitophagy abrogates cancer cell proliferation *via* inducing oxidative stress (Boyle et al., 2018; Shen et al., 2018). On the other hand, cancer cells can utilize mitophagy to adapt particular metabolic stress, leading to therapy resistances (Ferro et al., 2020). A previous study showed that the loss of PPARγ in platelet is closely associated with mitophagy activation, resulting in increased mitochondrial electron transport chain complex activity and enhanced mitochondrial ROS production (Zhou et al., 2017). However, the function and underlying mechanism of PPARγ-associated mitophagy during PDAC development remains largely unknown. Here, we aim to investigate the role of PPARγ on mitophagy and its involvement in the progression of PDAC.

## Methods

### Oncomine and TCGA Database Using

Expressions of PPARγ in normal pancreas and PDAC tissues were collected and analyzed using datasets deposited in Oncomine database (https://www.oncomine.org). The Cancer Type was defined as Pancreatic cancer and Data Type was mRNA, and Analysis Type was Cancer vs. Normal Analysis.

The expression levels of PPARγ, SOD2, ATG4D and survival data for TCGA pancreatic adenocarcinoma (provisional) patients (*n* = 176) were downloaded from The Human Protein Atlas (https://www.proteinatlas.org/). The best cutoff value for PPARγ, SOD2 and ATG4D mRNA expression level (FPKM) was 3.64, 11.98 and 6.65 respectively.

### Human PDAC Tissue Array Analysis

The study was conducted in accordance with International Ethical Guidelines for Biomedical Research Involving Human Subjects (CIOMS). The clinical part of study was approved by the Research Ethics Committee of Drum Tower Hospital, School of Medicine, Nanjing University. The patient cohort of human PDAC tissue array contained 59 PDAC specimens from May 2004 to November 2016 obtained from Drum Tower Hospital (School of medicine, Nanjing University). Patients had not received radiotherapy, chemotherapy or other related anti-tumor therapies before surgery.

### Immunohistochemistry

IHC of PDAC tissues was performed as described previously (Nie et al., 2020). Specific antibodies used for immunohistochemistry were: PPARγ (1:200, Proteintech, #16643-1-AP). PPARγ was localized mainly (but not always) in the nucleus. Only the staining of nucleus was counted and analyzed in this study, which revealed the prognostic effect of PPARγ on PDAC patients. The proportion of nucleus stained was evaluated as follows: 0 for <5%, 1 for 5–25%, 2 for 25–50%, 3 for 50–75%, and 4 for ≥ 75%. The intensity of staining was scored as 0, 1, 2 and 3 for the representation of no color, yellow, brown and dark brown. The final scores were obtained by multiplying the extent of positivity and intensity scores. Final score ≥ 3 was defined as positive. The stained slides were evaluated by two experienced pathologists independently.

### Cell Culture and Reagents

Human pancreatic cancer cell lines AsPC-1, BxPC3, Capan2, CFPAC-1, HPAC, MIAPaCa-2, PANC-1, SW1990 were gifts from Klinikum rechts der Isar, Technical University of Munich. All cell lines were cultured in suggested medium according to ATCC protocols. HPAC, SW1990 and PANC-1 cell lines were cultured with DMEM (Bio-Channel).

Rosiglitazone (APExBIO, #A4303), a therapeutic drug for diabetes, was used as an agonist of PPARγ. T0070907 (Selleck Chemicals, #S2871) was used as an antagonist of PPARγ. Cells were treated with Rosiglitazone (0, 10 or 20 μM) or T0070907 (0, 5 or 10 μM) for 72 h 10μM Chloroquine (CQ) (MedChemExpress, #HY-17589), gemcitabine (MedChemExpress, #HY-17026) was used for research.

### Luciferase Reporter Assay

The PPAR response element (PPRE) X3-TK-Luc plasmid is a reporter construct containing three copies of PPRE (PPRE X3) upstream of a thymidine kinase (TK) promoter fused to a luciferase gene (Tsai et al., 2014). The DNA sequence of this commercialized plasmid was downloaded from https://www.addgene.org. (Addgene, #1015) and constructed by Genechem (Shanghai, China). The plasmid was transfected into HPAC and SW1990 cells after treated with Rosiglitazone (0, 10 or 20 μM) or T0070907 (0, 5 or 10 μM) for 48 h. After another 24 h, the luciferase Assay System Kit (E1910, Promega, United States) was used to detect PPRE-driven luciferase activity.

### RNA Extraction and Quantitative Real-Time PCR

Total RNA was isolated using Trizol reagent (Takara) according to the manufacturer’s instructions. Reverse transcription reactions and Quantitative PCR were carried out as described previously. Reactions were run in triplicate in three independent experiments. The 2^−ΔΔCT^method was used to determine the relative levels of mRNA expression between experimental samples and controls. Primers were listed as following: ATG4A forward: TTC​CCT​TGA​GTG​CTG​ACA​CA; ATG4A reverse: ATT​TGG​TTT​ATG​CCC​AGG​CG. ATG4B forward: CAC​CAG​ATA​GCG​CAA​ATG​GG; ATG4B reverse: CTC​CAC​GTA​TCG​AAG​ACA​GCA. ATG4C forward: TGG​ACT​TCC​CAC​ACT​GTC​AAA; ATG4C reverse: AGG​GGG​AAT​CAC​CAA​ACC​AA; ATG4D forward: 5′-TGG​TGT​ACG​TTT​CTC​AGG​ACT-3′; ATG4D reverse: 5′-CAC​ATA​CAC​GGG​GTT​GAG​AGT-3′. SOD2 forward: GTC​AAC​CAT​CAA​AGA​GGT​CTG​C; SOD2 reverse: GAC​TGG​AGA​TAC​AGG​TCT​TGG​T. β-actin forward: 5′-CTA​CGT​CGC​CCT​GGA​CTT​CGA​GC-3′; β-actin reverse: 5′-GAT​GGA​GCC​GCC​GAT​CCA​CAC​GG-3′. CD24 forward: CAT​GGG​CAG​AGC​AAT​GGT​G; CD24 reverse: TAG​TTG​GAT​TTG​GGG​CCA​ACC. CD44 forward: TAC​AGC​ATC​TCT​CGG​ACG​GA; CD44 reverse: GCA​GGT​CTC​AAA​TCC​GAT​GC. CD 90 forward: GCA​GAA​GGT​GAC​CAG​CCT​AA; CD90 reverse: TGG​TGA​AGT​TGG​TTC​GGG​AG. CD133 forward: CAC​TAC​CAA​GGA​CAA​GGC​GT; CD133 reverse: TCC​AAC​GCC​TCT​TTG​GTC​TC. ESA forward: CTG​GCC​GTA​AAC​TGC​TTT​GT; ESA reverse: AGC​CCA​TCA​TTG​TTC​TGG​AGG.

### Western Blot

Cell and tissues lysates were collected as previously described (Nie et al., 2020). Protein concentrations were determined using BCA Assay (Beyotime Biotechnology). Equal amounts of protein were separated with 8–12% SDS-PAGE and then electrophoretically transferred onto a polyvinylidene difluoride membrane (Millipore, Billerica, MA, United States). TBST containing with 5% nonfat milk or bovine serum albumin was used to block nonspecific binding for 2 h at room temperature. Then, membranes were incubated with primary antibodies according to the instructions overnight at 4°C followed by appropriate secondary antibodies. Signals generated by enhanced chemiluminescence (Millipore) were recorded with a CCD camera (Tanon, Shanghai). Primary and secondary antibodies included: PPARγ (1:1,000, Santa cruze, #sc-7273), BCL-XL (1:1,000, Cell Signaling Technology, #2764), BAX (1:1,000, Cell Signaling Technology, #5023), BNIP3 (1:500, Cell Signaling Technology, #44060), LC3B (1:1,000, Abcam, #ab51520), P62 (1:1,000, Abcam, #ab109012), mTOR (1:1,000, Cell Signaling Technology, #2983), p-mTOR^Ser2448^ (1:1,000, Cell Signaling Technology, #5536), ULK1 (1:1,000, Cell Signaling Technology, #6439), p-ULK1^Ser317^ (1:1,000, Cell Signaling Technology, #12753), S6 (1:1,000, Cell Signaling Technology, #2217), P-S6^Ser235/236^ (1:1,000, Cell Signaling Technology, #2211), ATG4D (1:400, Zen-bioscience, #507842), SOD2 (1:1,000, proteintech, #66474-1-Ig), GAPDH (1:5,000, Proteintech, #60004-1-Ig), CD44 (1:1,000, Cell Signaling Technology, #5640), CD133 (1:1,000, Sigma-Aldrich, #4300882).

### Cell Proliferation Analysis

HPAC, SW1990 and PANC-1 cells were plated into 96-well plates at a concentration of 10^3 cells per well in 100 μL complete growth medium. Rosiglitazone (0, 10 or 20 μM) or T0070907 (0, five or 10 μM) was added to the cells 24 h after seeding. Cell viability was analyzed 1, 2, 3 and 4 days after cell seeding with Cell Counting Kit-8 (Dojindo, Kumamoto, Japan) according to the manufacturer’s instructions. Gemcitabine (0, 1, 2, 5, 10, 20, 50, 100, 200 μM) combined with 10 μM Rosiglitazone or not was added to the cells 24 h after seeding. Cell viability was analyzed 3 days after cell seeding with Cell Counting Kit-8 (Dojindo, Kumamoto, Japan) according to the manufacturer’s instructions.

### Colony-Formation Assay

Cells were plated in six-well plates in 2 ml complete medium with Rosiglitazone (0, 10 or 20 μM) or T0070907 (0, 5 or 10 μM). Numbers of cells per well were 1,000 for HPAC and SW1990 cells. The culture media with reagent was replaced by complete medium (2 ml) 4 days after cell seeding. After 14 days, colonies were fixed in methanol and stained with 0.5% crystal violet. The number of colonies was calculated by ImageJ.

### *In Vivo* Tumor Xenograft Study

Five-week-old male BALB/c nu/nu mice were purchased from CAVENS lab animal corporation. HPAC and PANC-1 cells were inoculated subcutaneously (1 × 10^6^ cells) into the left flank of each mouse. Six days after inoculation of HPAC, 14 days after inoculation of PANC-1, the mice were randomly divided into three groups and treated with Rosiglitazone (100 mg/kg) or T0070907 (5 mg/kg) *via* gavage three times a week. The tumor volumes were measured and calculated by the following formula (A*B^2^)/2, where A is the length and B is the width of the two dimensions of tumor. After animals were sacrificed, the weights of the tumor mass were measured.

### Mitochondria ROS Detection by Flow Cytometry

Mitochondria ROS (mito-ROS) was measured by using MitoSOX red (Yeasen, 40778ES50) according to the manufacturer’s instructions, at a concentration of 2 μM and incubated at 37°C with 5% CO_2_ for 20 min. Quantification of mito-ROS was carried out by flow cytometry.

### JC-1 Analysis for Mitochondrial Membrane Potential

Mitochondrial membrane depolarization was monitored by changes in the tetraethyl-benzimidazolylcarbocyanine iodide (JC-1) (Beyotime, C2006) green: red fluorescence ratio, where an increased ratio is indicative of elevated mitochondrial membrane potential (MMP). And the increased ratio could be a landmark of the early stage of apoptosis. Cells were incubated with JC-1 (1:1,000 dilution) for 20 min at 37°C. Then cells were harvested, washed twice with 1 × washing buffer and mixed in 100 μL of 1 × washing buffer. The fluorescence intensity was measured by flow cytometry.

### Flow Cytometric Analysis for Apoptosis

Cells with various culture reagent were seeded in 6-well plates. Cells were harvested, washed twice with PBS, and mixed in 100 μL of 1*binding buffer. After culturing in Annexin-V/PI (BD Biosciences) double staining liquid for 15 min at room temperature in the dark, the cells were examined by flow cytometry.

### Mitophagy Assay

For quantification of mitophagic flux, cells were treated with Mtphagy dye (Mitophagy Detection Kit, Dojindo Molecular Technologies, #MD01) according to the manufacturer’s instruction. The fluorescence intensity of Mtphagy dye (excitation 530 nm; emission 700 nm) was measured by flow cytometry. Increased Mtphagy dye fluorescence intensity indicated the progression of mitophagy.

### Immunofluorescence

Cells treated with different reagents were seeded in 24-well plates at a density of 1 × 10^3^ cells per well. At 72 h after seeding, cells were fixed with 4% paraformaldehyde for 15 min at room temperature and permeabilized with 0.2% Triton X-100 for 15 min. After blocking with 5% BSA for 1 h at room temperature, cells were incubated with LC3B (1:100, Cell Signaling Technology, #3868), TOM20 (1:200, Santa cruze, #17764) or ATG4D (1:100, Zen-bioscience, #507842) antibodies overnight at 4°C. Cells were washed 3 times and then incubated with Alexa Fluor^®^ 488 (Abcam, ab150077) or Alexa Fluor^®^ 647 (Abcam, 150115) for 1 hour at room temperature in the dark. Then, cells were incubated with DAPI (Beyotime, C1005) for 20 min. Cells were visualized by a fluorescence microscope (Olympus, Tokyo, Japan).

### Electron Microscopy

After collecting cells for different treatment, cells were centrifuged and pellets were fixed with 0.1 M cacodylate buffer with a pH of 7.4 at RT and sections were processed by the Electron Microscopy unit per standard protocols. Pictures were taken with a Hitachi transmission electron microscope (TEM) system.

### Chromatin Immunoprecipitation Assay

The chromatin immunoprecipitation (ChIP) assay was performed as instructions from Magna ChIP Kit (Merck, #17-10085). Precleared cell lysates were incubated with the antibodies at 4°C overnight. Immune complexes were recovered with salmon sperm DNA/protein A/G agarose slurry. After washing and elution, genomic DNA was extracted with phenol/chloroform for PCR analysis. The PCR primer of SOD2 was following. Forward: 5′- AGT​ACC​TCC​TGC​TGA​GAC​GA- 3′. Reverse: 5′- TGG​GAA​AAC​AGT​CAG​GCG​AA- 3′.

### siRNA Transfection

Cells were transfected with either two siRNAs against SOD2, ATG4D or one non-targeting siRNA and cultured in 6-well plates according to the manufacturer’s instructions. The target sequences of oligo siRNAs were as follows: siPPARγ#1: forward: ACU​CCA​CAU​UAC​GAA​GAC​ATT, reverse: UGU​CUU​CGU​AAU​GUG​GAG​UTT. siPPARγ#2: forward: CUG​GCC​UCC​UUG​AUG​AAU​ATT, reverse: UAU​UCA​UCA​AGG​AGG​CCA​GTT. siSOD2#1: forward: CUG​GGA​GAA​UGU​AAC​UGA​A, reverse: UUC​AGU​UAC​AUU​CUC​CCA​G. siSOD2#2: forward: CAC​GCU​UAC​UAC​CUU​CAG​U, reverse: ACU​GAA​GGU​AGU​AAG​CGU​G. siATG4D#1: forward: GGC​AGA​UUG​UGU​CCU​GGU​UTT, reverse: AAC​CAG​GAC​ACA​AUC​UGC​CTT. siATG4D#2: forward: GGA​AGG​AGU​UUG​AGA​CAC​UTT, reverse: GGA​AGG​AGU​UUG​AGA​CAC​UTT. Negative control: forward: UUC​UCC​GAA​CGU​GUC​ACG​UTT, reverse: UUC​UCC​GAA​CGU​GUC​ACG​UTT.

### Sphere-Formation Assay

HPAC cell spheres were generated and expanded in DMEM-F12 (Invitrogen) supplemented with 20 nM epidermal growth factor (EGF) (Sigma-Aldrich, #E4127), 20 nM basic fibroblast growth factor (bFGF) (Gibco, #aa1-155) and 3% FBS. One thousand cells/ml/well were seeded in ultra-low attachment 24-well plates as described previously (Gallmeier et al., 2011). For serial passaging, spheres were harvested at day 7 using a 40-µm cell strainer, dissociated to single cells with trypsin, and then re-grown in the same conditions for 7 days (14 days total). Spheres were defined as morphologically characteristic three-dimensional structures of approximately ≥ 75 μm (Mani et al., 2008). Diameters and numbers of spheres were determined by an inverted microscope (Thermo Fisher Scientific) using a ×10, ×20 objective with phase contrast.

### Statistical Analyses

Data were analyzed using GraphPad Prism v7.0. All experiments were repeated at least three times, with the mean and standard deviation (S.D.) being reported where appropriate. The repeated results were used as data points for statistical tests. Differences between treatments were evaluated using ANOVA or Student’s *t* test. Correlations were analyzed by the Pearson method. Log-rank tests were performed on Kaplan-Meier survival curves to determine any significant relationships between gene expression and patient outcomes. Differences were considered significant at *p* < 0.05.

## Results

### High Expression of PPARγ in Nucleus is Correlated With Poor Prognosis in PDAC

**FIGURE 1:**
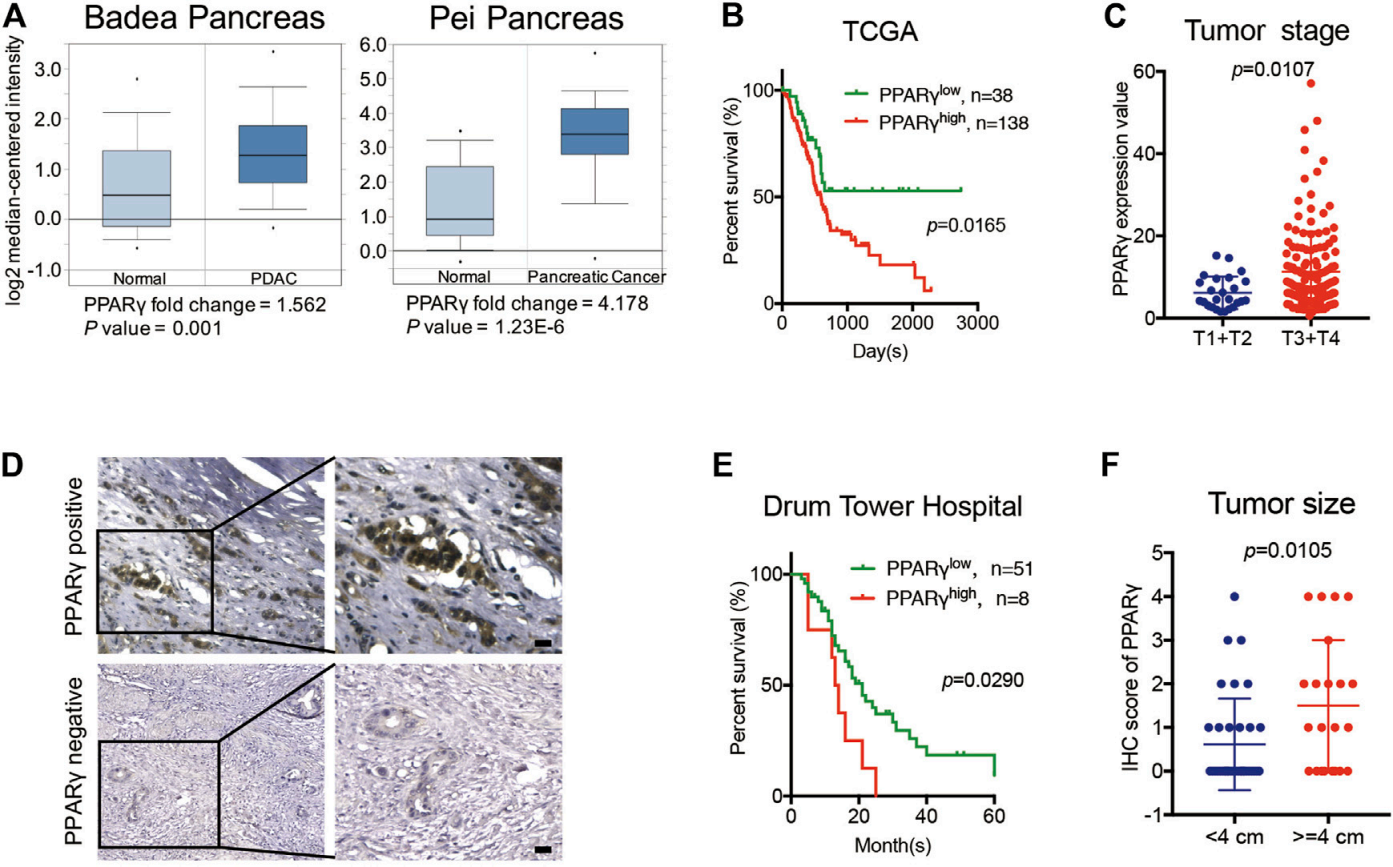
High expression of PPARγ in nuleus is correlated with poor prognosis in PDAC. **(A)** The expression of PPARγ increased in pancreatic cancer tissue compared to adjacent normal pancreatic tissue by Oncomine datasets analysis. **(B)** In TCGA dataset, high expression of PPARγ in cancer tissues was associated with shorter overall survival time in the PDAC patients (*p =* 0.0165). **(C)** In TCGA dataset, PPARγ expression level was positively correlated with tumor stages (*p =* 0.0107). **(D)** PPARγ immunostaining signal in pancreatic cancer tissues was primarily detected in nucleus. The scale bar was 20 μm. **(E)** High expression level of PPARγ in pancreatic cancer tissues was associated with shorter overall survival time in the PDAC patients (*p =* 0.0290). **(F)** PPARγ expression level was positively correlated with tumor size (*p =* 0.0105).

Two previously published datasets from Oncomine were initially analyzed to determine the expression pattern of PPARγ in pancreatic cancer and normal tissues. The results revealed that PPARγ expression level was upregulated in cancer tissues compared to that in the corresponding adjacent non-tumor tissues (Figure 1A). We then analyzed TCGA dataset and found that the mRNA expression of PPARγ was correlated with overall survival and tumor stage. PDAC patients with high PPARγ expression exhibited advanced tumor stages and poor prognosis compared to those with low PPARγ expression (Figures 1B,C).

To further examine the expression and clinical relevance of PPARγ in PDAC, we detected the expression of PPARγ in 59 human PDAC tissues from Nanjing Drum Tower Hospital. Immunohistochemical staining on tissues was performed, and the results showed that the immunostaining signal of PPARγ was mainly located in nucleus (Figure 1D) and only the expression level of nuclear PPARγ was correlated with overall survival. Kaplan-Meier analysis revealed that the positive expression of nuclear PPARγ in cancer tissues was associated with short overall survival time in PDAC patients (*p* = 0.0290) (Figure 1E). In addition, the nuclear PPARγ expression level was significantly related to tumor size (*p* = 0.0105) (Figure 1F).

### PPARγ Promotes Pancreatic Cancer Cells Proliferation *in Vitro* and *in Vivo*

**FIGURE 2:**
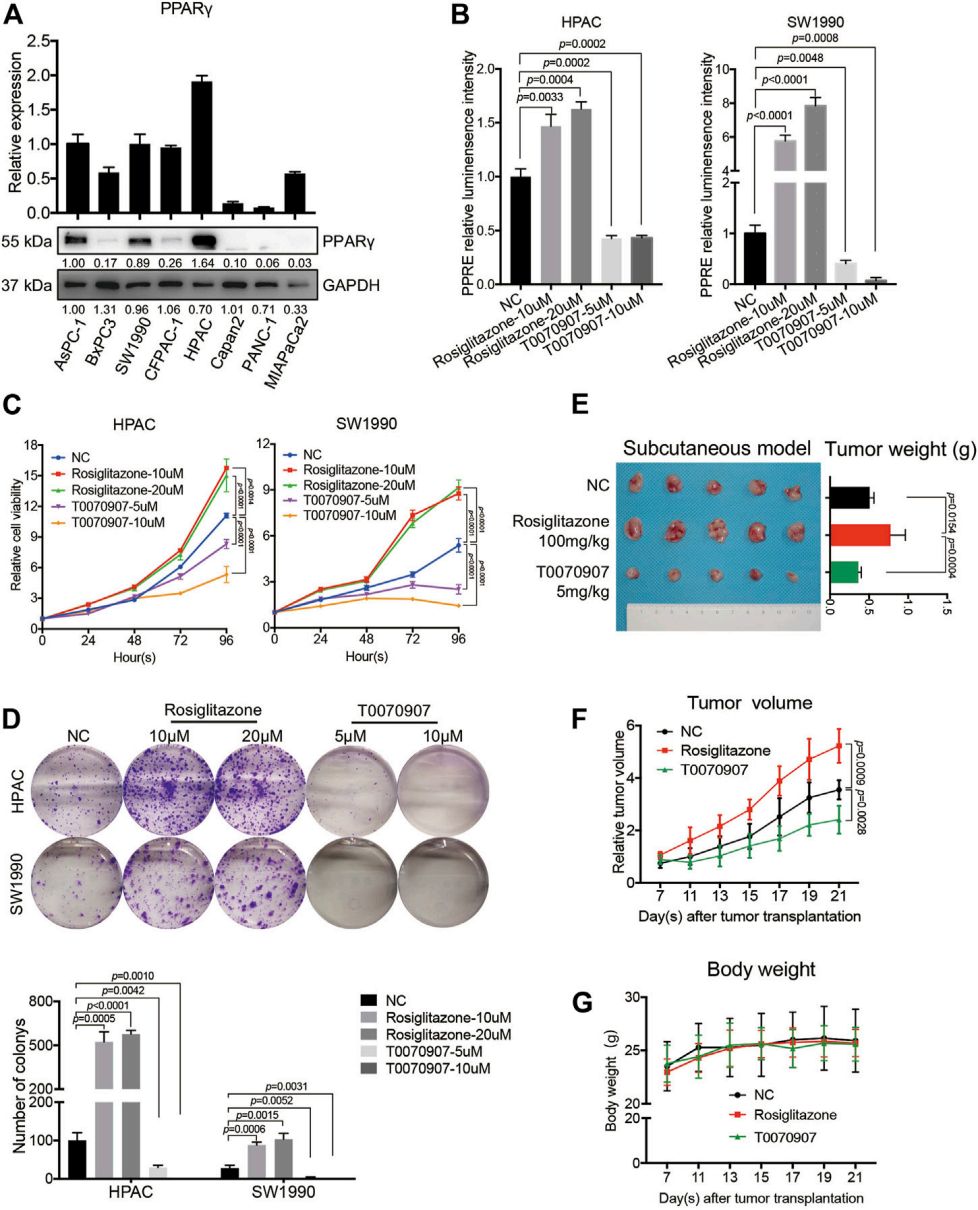
PPARγ promotes pancreatic cancer cell proliferation *in vitro* and *in vivo.* **(A)** The expression status of PPARγ was detected in eight human pancreatic cancer cell lines at mRNA and protein levels. **(B)** The PPRE-driven luciferase reporter gene assay showed Rosiglitazone could activate, while T0070907 could inhibit PPARγ transcriptional activity. **(C)** Relative cell viability of HPAC and SW1990 cells treated with negative control, Rosiglitazone (10, 20 μM) or T0070907 (5, 10 μM) for 0, 24, 48 and 72 h. **(D)** Formation of colonies of HPAC and SW1990 cells treated with negative control, Rosiglitazone (10, 20 μM) or T0070907 (5, 10 μM) for 72 h **(E–G)** Compared with the control group, administrating nude mice with Rosiglitazone (100 mg/kg) or T0070907 (5 mg/kg) reduced or increased the tumor weight **(E)** and the tumor volume **(F)** 4 weeks after HPAC cells injection, without the influence on body weight of mice **(G)**. Experiments were repeated at least three times, with statistical analyses being reported appropriate.

To investigate the biological functions of PPARγ in PDACs, human PDAC cell lines were used for further studies. Firstly, the mRNA and protein expression levels of PPARγ were detected in different human pancreatic cancer cell lines. HPAC and SW1990 cells with positive expression of PPARγ were selected for subsequent study (Figure 2A).

PPARγ is a member of the nuclear receptor superfamily that functions as a ligand-activated transcription factor (Tseng et al., 2019). Here, we used PPARγ agonist Rosiglitazone or PPARγ antagonist T0070907 to intervene the PPARγ pathway and to investigate whether pharmacological activation or inhibition of PPARγ would affect the cancer cell survival. To confirm the effects of agonist or antagonist on the transcriptional activity of PPARγ, PPRE-driven luciferase activity was detected in HPAC and SW1990 treated with Rosiglitazone, T0070907 or not. The results showed that Rosiglitazone could enhance, while T0070907 could weaken the transcriptional activity of PPARγ significantly (Figure 2B). *In vitro* CCK8 assay and colony-formation assay results showed that Rosiglitazone could promote, while T0070907 could inhibit the proliferation and colony-formation capacity of HPAC and SW1990 cells (Figures 2C,D), which was further confirmed in nude mice models. After injecting 1 × 10^6^ HPAC cells into nude mice to construct tumors *in vivo,* solvent or Rosiglitazone (100 mg/kg) or T0070907 (5 mg/kg) was given to treat mice three times a week, respectively. Results revealed that Rosiglitazone promoted, while T0070907 inhibited tumor growth without influencing the weight of nude mice (Figures 2E–G).

### PPARγ Inhibits Mitochondrial ROS-Dependent Apoptosis in Pancreatic Cancer Cells

**FIGURE 3:**
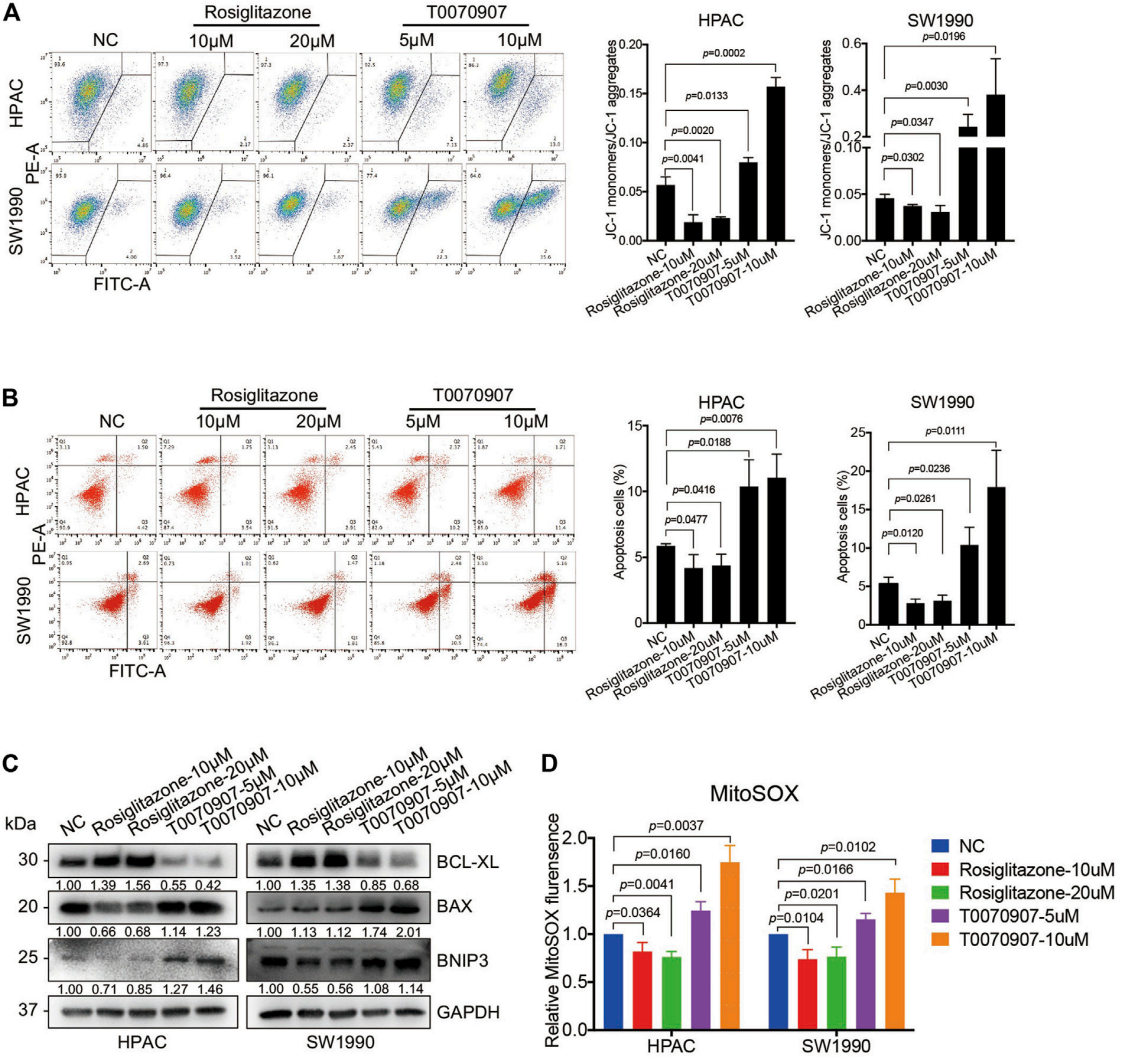
PPARγ inhibits mitochondrial ROS-dependent apoptosis in pancreatic cancer cells. **(A)** Treating HPAC or SW1990 cells with negative control, Rosiglitazone (10, 20 μM) or T0070907 (5, 10 μM) for 72 h affected mitochondrial membrane potential (MMP) determined by JC-1 fluorescent intensity. **(B)** Treating HPAC or SW1990 cells with negative control, Rosiglitazone (10, 20 μM) or T0070907 (5, 10 μM) for 72 h affected cell apoptosis determined by flow cytometry with Annexin V-FITC/PI staining. **(C)** The molecular changes in apoptosis determined by Western blot with antibodies against BCL-XL, BAX and BNIP3. **(D)** Treating HPAC or SW1990 cells with negative control, Rosiglitazone (10, 20 μM) or T0070907 (5, 10 μM) for 72 h affected mitochondrial ROS levels determined and quantitated by MitoSOX staining. Experiments were repeated at least three times, with statistical analyses being reported appropriate.

To detect the mechanisms underlying the role of PPARγ on pancreatic cancer cell survival, we determined the early stage of apoptosis by JC-1 assay in the presence or absence of Rosiglitazone or T0070907. An increase in green fluorescence and the concomitant damage of red fluorescence (increased green: red ratio) were observed in cells treated with T0070907, and an inversed ratio was observed in cells treated with Rosiglitazone, indicating the effect of PPARγ on maintaining the mitochondrial membrane potential (MMP) (Figure 3A). To further assess the apoptotic rate upon drug treatment, flow cytometry analysis was performed after FITC Annexin V/PI staining, which showed that PPARγ significantly protected against pancreatic cancer cell apoptosis (Figure 3B). In addition, there were increased levels of pro-apoptotic factors—BAX and BNIP3 in HPAC and SW1990 cells treated with T0070907, coupled with decreased level of anti-apoptotic factor BCL-XL, and it showed the opposite results in cells treated with Rosiglitazone (Figure 3C). These data suggested that the activation of PPARγ could effectively promote PDAC proliferation *via* inhibiting pancreatic cancer cell apoptosis.

Elevated intracellular levels of ROS induces oxidative stress, leading to cell death. In view of the potential function of PPARγ on mitochondrial metabolism, the MitoSOX red fluorescent dye was used to detect the effect of PPARγ on mitochondria-originating ROS alterations. This assay showed a significant decrease of fluorescence in cells with Rosiglitazone treatment, and an increase in cells with T0070907 treatment (Figure 3D).

Besides, to confirm the influence of PPARγ agonist-Rosiglitazone or antagonist-T0070907 was in a PPARγ-dependent way, PANC-1 cell line with PPARγ-negative expression was chosen for *in vitro* functional assays as well. Though the statistical analysis results showed that T0070907 could inhibit cell proliferation after 96 h treatment *in vitro*, it had no effect on cell growth and the body weight of mice *in vivo* (Supplementary Figures S1A–D). Besides the results showed that only 10 μM T0070907 could induce increased mitochondria membrane potential and apoptosis, while Rosiglitazone and low-dose of T0070907 had no effect on mitochondrial ROS production, mitochondria membrane potential and apoptosis in PANC-1 cells (Supplementary Figures S1E–G). We supposed that high-dose of T0070907 might induce apoptosis *via* oxidative stress in a PPARγ-independent manner (Kawahara et al., 2013). Thus, in HPAC and SW1990 cells (with PPARγ-positive expression), the growth-promoting role of Rosiglitazone or -inhibiting role of T0070907 were primarily and specifically dependent on PPARγ.

Additionally, the gene-knockdown assay in HPAC and SW1990 cell lines was performed to further verify the function of PPARγ in regulating tumor cell survival. The knockdown efficiency of PPARγ in HPAC and SW1990 cells was detected both on mRNA and protein levels (Supplementary Figure S2A). The transcriptional activity of PPARγ was impaired after interfering PPARγ expression (Supplementary Figure S2B). Moreover, there were elevated levels of pro-apoptotic factors—BAX and BNIP3, depressed level of anti-apoptotic factor BCL-XL in PPARγ-knockdown cells compared to negative control cells (Supplementary Figure S2C). And the results of flow-cytometry also revealed that PPARγ-knockdown could contribute to increased mitochondrial membrane potential (Supplementary Figure S2D) and apoptosis (Supplementary Figure S2E) in HPAC and SW1990 cells.

### PPARγ Regulates the mTOR-ULK1 Signaling Pathway to Inhibit Mitophagy in Pancreatic Cancer Cells

**FIGURE 4:**
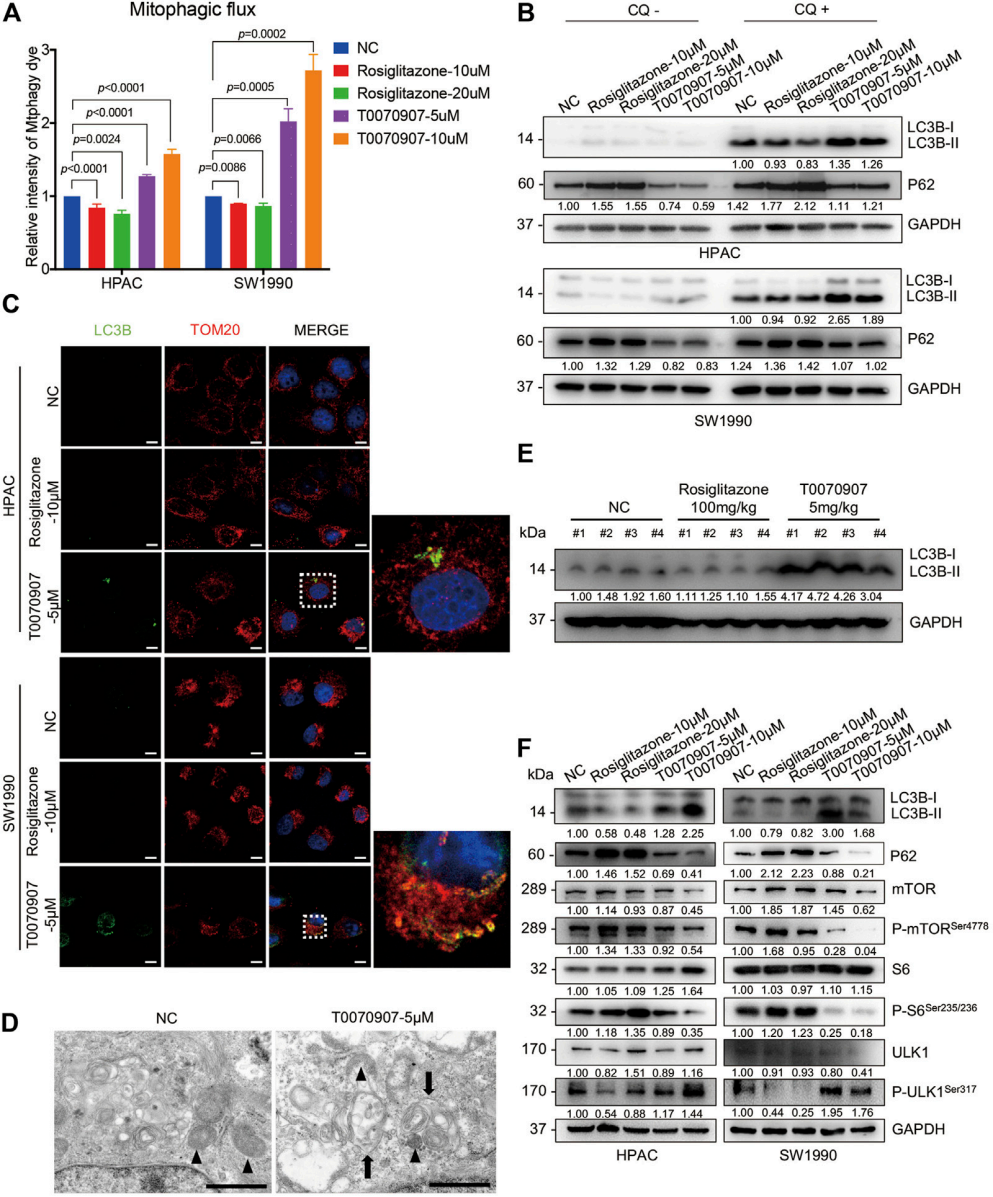
PPARγ regulates the mTOR-ULK1 signaling pathway to inhibit mitophagy in pancreatic cancer cells. **(A)** The inhibition of PPARγ activated mitophagic flux detected by flow cytometry in HPAC and SW1990 cells. **(B)** Treating HPAC or SW1990 cells with negative control, Rosiglitazone (10, 20 μM) or T0070907 (5, 10 μM) in the absence or presence autophagy inhibitor CQ (10 μM), LC3B-II and P62 was detected by Western blot to indicate the autophagic flux. **(C)** The effects of T0070907 on mitophagy indicated by LC3B-II expression was further confirmed by cell Immunofluorescence. The scale bar was 20 μm. **(D)** Representative TEM graphs showed the existence of mitophagosome after T0070907 treatment in HPAC cells. Black triangle: mitochondria. Black arrow: autophagosome. The scale bar was 10 μm. **(E)** The protein expression level of LC3B-II was elevated in the mice tumor tissues treated with T0070907 compared to negative control group. **(F)** Treating HPAC or SW1990 cells with negative control, Rosiglitazone (10, 20 μM) or T0070907 (5, 10 μM) for 72 h changed the mTOR/ULK1 signaling pathway. Experiments were repeated at least three times, with statistical analyses being reported appropriate.

Mitophagy has crucial effects on controlling mitochondrial quality and function (Pickles et al., 2018). To precisely assess the effect of PPARγ on mitophagy, we quantified mitophagic flux using Mtphagy dye after treating cells with T0070907. Mtphagy dye stains mitochondria, and its fluorescence intensity depends on the pH. When mitochondria are transported to lysosomes by mitophagy, Mtphagy dye exhibits higher fluorescence intensity. We found that the activation of PPARγ by Rosiglitazone, or the inhibition of PPARγ by T0070907 in HPAC and SW1990 cells could inhibit, or activate the mitophagic flux, respectively (Figure 4A). The activation of different steps of autophagy (the autophagosome formation or the lysosome-autophagosome fusion) would lead to the dynamic change on LC3B-II expression level. Since p62 accumulates when autophagy is inhibited, and decreased levels can be observed when autophagy is induced, p62 is used as a marker to study autophagic flux (Bjørkøy et al., 2009). Thus, the expression level of autophagy marker was further confirmed by treating cells with chloquine, an inhibitor of lysosome function. We found that there was no difference in trends of expression level of P62 in Rosiglitazone- and T0070907-treated groups with or without CQ, that Rosiglitazone increasing and T0070907 decreasing the expression level of P62 (Figure 4B). Furthermore, under the condition of lysosomal degradation-blocking, LC3B-II, another hallmark of autophagy activation, accumulating significantly in T0070907-treated cells compared to negative control group cells. The above results revealed that PPARγ had a strong effect on autophagic flux (Figure 4B). Results from cell immunofluorescence also showed the green fluorescent signal of LC3B increased in T0070907-treated cells and it co-localized with the red fluorescent signal of TOM20, a mitochondrial outer membrane protein (Figure 4C), indicating the occurrence of mitophagy. Evidence of T0070907-induced mitophagy in HPAC cells was determined by direct observation of the formation of mitophagosomes using electron microscopy (Figure 4D). Additionally, the protein expression level of LC3B-II in nude mice transplanting tumors treated with T0070907 was elevated significantly as well (Figure 4E).

The mTOR pathway commonly participates in autophagic process. On one hand, mTOR could contribute to regulating termination of autophagy and reformation of lysosomes (Yu et al., 2010). On the other hand, mTOR could inhibit the autophagosome formation *via* ULK1 ubiquitylation (Nazio et al., 2013). The mammalian orthologue of yeast Atg1, the serine/threonine kinase ULK1, plays a key role in autophagy induction (Hosokawa et al., 2009). Thus, it prompts us to examine the alteration of mTOR pathway in cells treated with Rosiglitazone or T0070907. As shown, the phosphorylation levels of mTOR and S6 (the classical downstream target of mTOR) were increased in cells with Rosiglitazone treatment, while decreased in cells with T0070907 treatment (Figure 4F), indicating the activated process of regulating termination of autophagy and reformation of lysosomes. Unc-51 Like Autophagy Activating Kinase 1 (ULK1) regulates the initiation of autophagy by recruiting downstream autophagy-related proteins (ATGs) to autophagy formation site. And the phosphorylation of ULK1 would be inhibited by mTOR complex 1 (mTORC1) activation, thereby inhibiting the autophagy occurrence. Thus, we further determined the status of ULK1 in cells treated with Rosiglitazone or T0070907. The phosphorylation level of ULK1 markedly increased along with the decrease of phospho-mTOR in HPAC and SW1990 cells treated with T0070907 compared to negative control group cells, the situation of which was opposite in cells treated with Rosiglitazone (Figure 4F). In this part, we figured out that PPARγ might regulate mTOR-mediated degradation of ULK1, linked to impaired mitophagy in pancreatic cancer cells.

### PPARγ Downregulates ATG4D-Mediated Mitophagy to Inhibit Pancreatic Cancer Cell Apoptosis

**FIGURE 5:**
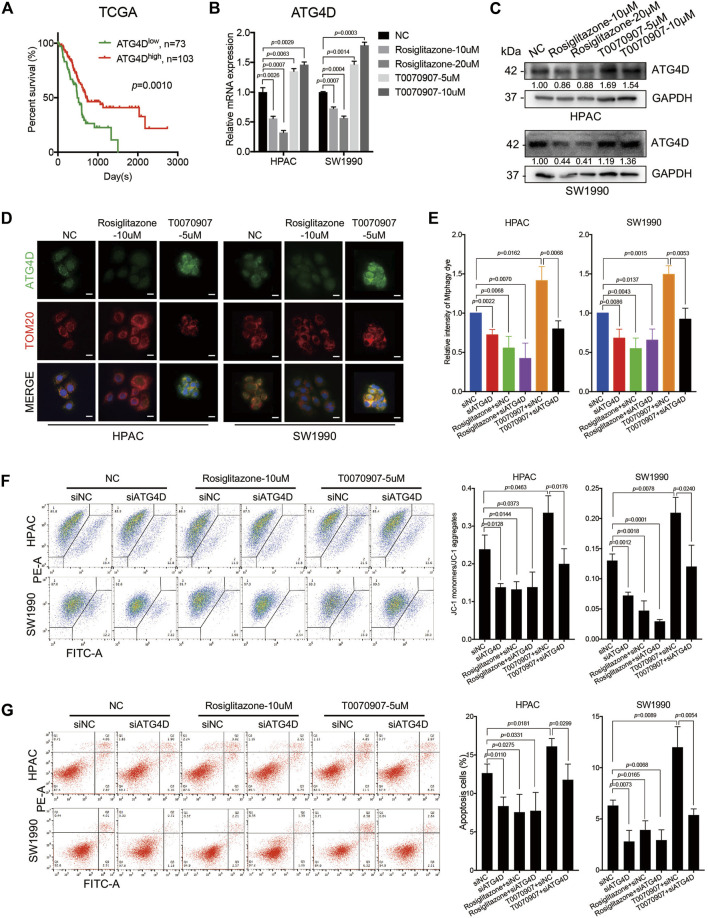
PPARγ downregulates ATG4D-mediated mitophagy to decrease mitochondrial ROS-dependent apoptosis. **(A)** In TCGA dataset, the mRNA expression level of ATG4D was negatively correlated with PDAC patients’ overall survival time (*p* = 0.0010). **(B)** Treating HPAC or SW1990 cells with negative control, Rosiglitazone (10, 20 μM) or T0070907 (5, 10 μM) for 72 h affected the mRNA expression level of ATG4D. **(C)** Treating HPAC or SW1990 cells with negative control, Rosiglitazone (10, 20 μM) or T0070907 (5, 10 μM) for 72 h affected the protein expression level of ATG4D. **(D)** The expression and location of ATG4D was mainly changed on mitochondria after treating HPAC or SW1990 cells with negative control, Rosiglitazone (10 μM) or T0070907 (5 μM) for 72 h. **(E)** Knockdown of ATG4D by siRNA in HPAC and SW1990 cells decreased mitophagic flux with or without Rosiglitazone (10 μM) or T0070907 (5 μM). **(F)** Knockdown of ATG4D by siRNA in HPAC and SW1990 cells stabilized mitochondrial membrane potential with or without Rosiglitazone (10 μM) or T0070907 (5 μM). **(G)** Knockdown of ATG4D by siRNA in HPAC and SW1990 cells decreased cell apoptosis with or without Rosiglitazone (10 μM) or T0070907 (5 μM). Experiments were repeated at least three times, with statistical analyses being reported appropriate.

ATG4D, one member of autophagy-related gene 4 (ATG4) family, is able to re-localize to damaged mitochondria, contributing to targeted mitophagy. Additionally, ATG4D could expose the BH3 domain allowing for interaction with BCL-2 family members and induction of cell apoptosis. Notably, mRNA expression level of ATG4D in TCGA dataset was positively correlated with the overall survival of PDAC patients (Figure 5A). The expression level of ATG4 isoforms (ATG4A, ATG4B, ATG4C and ATG4D) were all detected in HPAC and SW1990 cells treated with Rosiglitazone or T0070907, while only ATG4D was dose-dependent on PPARγ in HPAC and SW1990 cells (Supplementary Figure S3A, Figures 5B,C). Thus, we focused on detecting the role of ATG4D in cells with different PPARγ status. Results from cell immunofluorescence further confirmed that ATG4D mainly expressed on mitochondria when it was activated by T0070907 (Figure 5D). It suggested that ATG4D could be down-regulated by PPARγ to inhibit autophagy especially located on mitochondria.

Studies have found that mitochondrial ATG4D sensitizes cells to death in the presence of the mitochondrial uncoupler, CCCP. And during the mitochondrial clearance phase in differentiating primary human erythroblasts stably expressing ATG4D, these cells have elevated levels of mitochondrial ROS (Betin et al., 2012). To further figure out the role of ATG4D-mediated mitophagy on mitochondrial ROS production, mitochondrial membrane potential and cell apoptosis, the downregulation of ATG4D *via* siRNA was performed in HPAC and SW1990 cells treated with Rosiglitazone, T0070907 or not. Firstly, knockdown efficiency of ATG4D was confirmed on mRNA and protein levels (Supplementary Figures S3B,C), and ATG4D-knockdown did inhibit the expression of LC3B-II (Supplementary Figure S3C). Additionally, we quantified mitophagic flux using Mtphagy dye after treating cells with ATG4D knockdown, and the results showed that downregulating ATG4D could block the role of T0070907 on mitophagy activation to inhibit mitophagic flux (Figure 5E). Furthermore, after inhibiting the expression of ATG4D by siRNA in cells, mitochondrial membrane potential (Figure 5F) and cell apoptosis (Figure 5G) were all decreased, no matter whether Rosiglitazone or T0070907 was used to treat cancer cells or not. These results revealed that PPARγ inhibited mitophagy *via* regulating ATG4D, decreasing the mitochondrial ROS-dependent cell apoptosis.

### PPARγ Inhibits Mitochondrial ROS-ATG4D-Mediated Mitophagy *via* Upregulating SOD2

**FIGURE 6:**
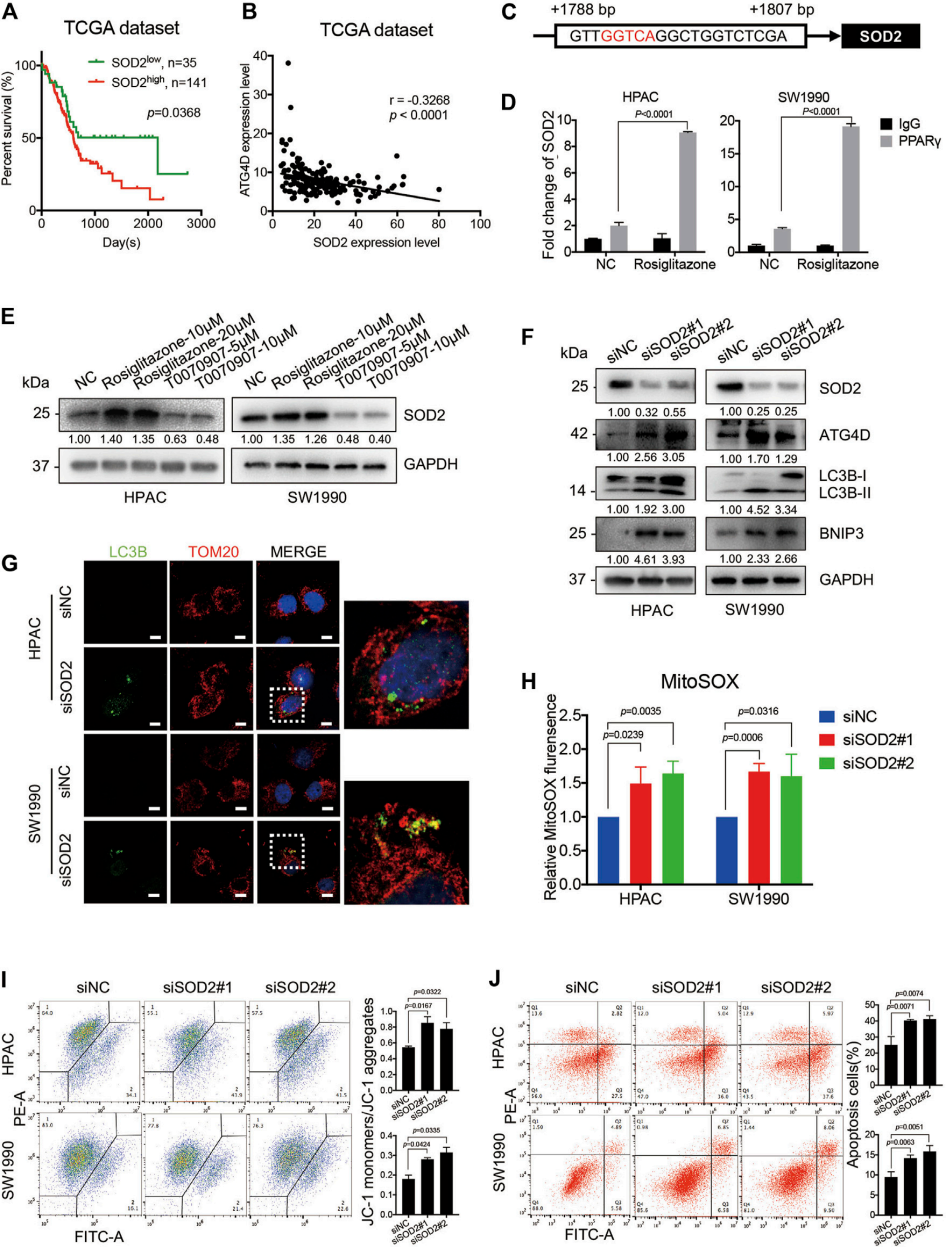
PPARγ inhibits ATG4D-mediated mitophagy *via* upregulating SOD2. **(A)** In TCGA dataset, The PDAC patients with SOD2 high expression suffered from shorter overall survival time than those with SOD2 low expression (*p* = 0.0368). **(B)** Analysis of TCGA data showed the expression of SOD2 and ATG4D had significant negative correlation (*r* =—0.3268, *p* < 0.0001). **(C)** The promoter regions of SOD2 contained PPAR response element (PPRE) (red letter). **(D)** ChIP assay confirmed that PPARγ regulated directly the transcription of SOD2 in HPAC and SW1990 cells. **(E)** Treating HPAC or SW1990 cells with negative control, Rosiglitazone (10, 20 μM) or T0070907 (5, 10 μM) for 72 h affected the expression level of SOD2. **(F)** Knockdown of SOD2 by siRNA in HPAC and SW1990 cells increased the expression of ATG4D, LC3B-II and the apoptosis-related protein expression-BNIP3. **(G)** Knockdown of SOD2 by siRNA in HPAC and SW1990 cells increased the expression of LC3B-II on mitochondria by cell immunofluorescence. The scale bar was 20 μm. **(H)** Knockdown of SOD2 by siRNA in HPAC and SW1990 cells increased mitochondrial ROS production. **(I)** Knockdown of SOD2 by siRNA in HPAC and SW1990 cells stabilized mitochondrial membrane potential. **(J)** Knockdown of SOD2 by siRNA in HPAC and SW1990 cells increased cell apoptosis. Experiments were repeated at least three times, with statistical analyses being reported appropriate.

SOD2, the primary mitochondrial oxidative scavenger, plays a crucial role during the regulation of mitochondrial ROS by catalyzing O2^−^ conversation to H_2_O_2_. The expression of SOD2 in TCGA dataset was negatively correlated to PDAC patients’ overall survival time (Figure 6A). Notably, the TCGA dataset revealed that there was a negative correlation between SOD2 and ATG4D mRNA expression level (Figure 6B). The effect of SOD2 expression on PPARγ-inhibiting mitochondrial ROS production was further investigated. *In silico* analysis predicted that the promoter region of SOD2 gene contained PPARγ binding sites, moreover one of which contained the PPRE binding site (Figure 6C). Consistently, ChIP assay results demonstrated that PPARγ could directly bind to the PPRE in the promoter region of *SOD2* in HPAC and SW1990 cells treated with Rosiglitazone (Figure 6D). The role of PPARγ on modulating SOD2 expression was also confirmed on the protein level (Figure 6E). To access if SOD2 could influence the ATG4D-mediated mitophagy, mitophagic markers, MitoSOX, JC-1 and cell apoptosis assays were detected after the inhibition of SOD2 by siRNA transfection. The blockade of SOD2 increased the protein expression level of ATG4D, as well as the accumulation of LC3B-II on mitochondria, the increased level of BNIP3 (Figures 6F,G).

Moreover, MitoSOX-based measurement revealed that, the inhibition of SOD2 by siRNA in HPAC and SW1990 cells could increase the mitochondrial ROS level (Figure 6H). Furthermore, SOD2 siRNA treatment also increase MMP as shown in JC-1 assay (Figure 6I) and cell apoptosis assay (Figure 6J) in HPAC and SW1990 cells. Taken together, these results suggested that PPARγ might inhibit ATG4D-mediated mitophagy *via* upregulating SOD2, to reduce mitochondrial ROS-dependent cell apoptosis.

The above results were also verified *via* PPARγ-knockdown assay in HPAC and SW1990 cells. The flow-cytometry result showed that PPARγ-knockdown in HPAC and SW1990 cells could activate mitophagic flux (Supplementary Figure S4A). And the expression of ATG4D and SOD2 was confirmed to be regulated by PPARγ in HPAC and SW1990 cells (Supplementary Figures S4B–D). Additionally, PPARγ knockdown in cells did influence mTOR-ULK1 pathway (Supplementary Figure S4D), to activate the expression of LC3B-II and induce mitophagy, which was also verified by cell immunofluorescence (Supplementary Figures S4D,E).

### PPARγ/SOD2 Decreases the Potential Stemness of PDAC *via* Inhibiting ATG4D-Mediated Mitophagy

**FIGURE 7:**
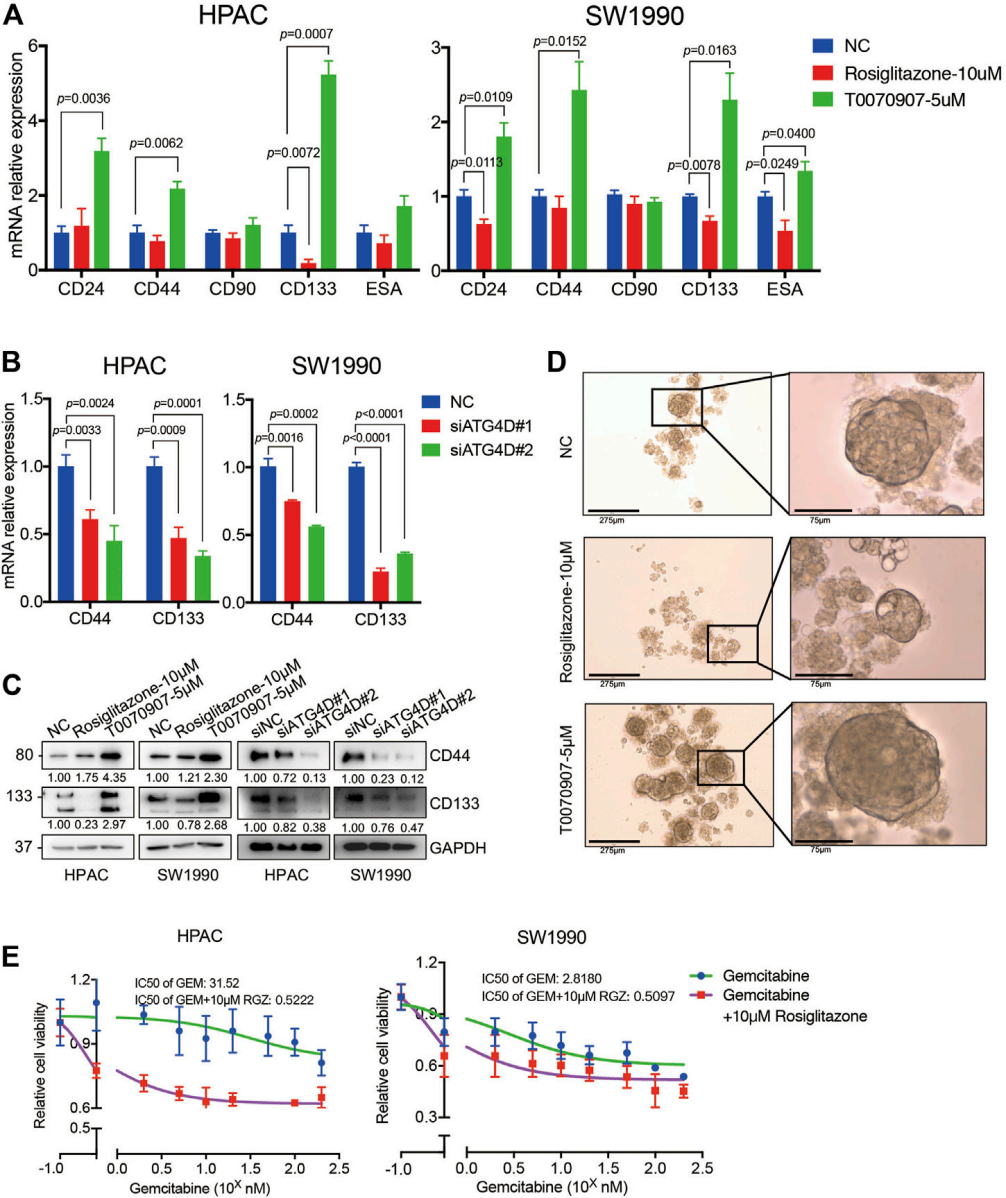
PPARγ inhibits the capacity of pancreatic cancer cell stemness and induces the sensitivity of Gemcitabine treatment. **(A)** Treating HPAC or SW1990 cells with negative control, Rosiglitazone (10 μM) or T0070907 (5 μM) for 72 h affected the CD44 and CD133 mRNA expression levels. **(B)** Knockdown of ATG4D by siRNA in HPAC and SW1990 cells decreased the CD44 and CD133 mRNA expression levels. **(C)** PPARγ activation or inhibition, or ATG4D downregulation affected CD44 and CD133 protein expression levels. **(D)** Rosiglitazone could promote, while T0070907 could inhibit the sphere-formation capacity of HPAC cells compared to negative control groups. **(E)** Combination of Rosiglitazone and Gemcitabine in HPAC and SW1990 cells significantly inhibited tumor cell viability compared with Gemcitabine alone. Experiments were repeated at least three times, with statistical analyses being reported appropriate.

Notably, pancreatic cancer stem cells (PaCSCs) could use mitophagy for particular adaptation of metabolic stress, contributing to better survive in tumor micro-environment (Ferro et al., 2020). Emerging evidence suggests that PaCSCs, marked by CD44, CD24, ESA, CD133, or c-Met proteins, characterize a subset of PDAC with distinct stemness features that permit them to drive tumor heterogeneity, metastasis and resistance to the current chemotherapy and radiation (Subramaniam et al., 2018). A previous study found that Rosiglitazone and Gemcitabine in combination reduces immune suppression in pancreatic cancer, participating in chemotherapy resistance (Bunt et al., 2013). To investigate whether the enhanced PDAC proliferation *via* PPARγ-inhibited mitophagic pathway has effects on stemness of pancreatic cancer cells and chemotherapy sensitivity, we detected the expression of PaCSCs’ markers. The results showed that Rosiglitazone could decrease, while T0070907 increase both the mRNA and protein expression levels of CD44 and CD133 in HPAC and SW1990 cells (Figure 7A). To confirm whether the effect of PPARγ on the expression of PaCSCs markers was dependent on ATG4D-mediated mitophagy, we detected the markers of PaCSCs expression after inhibiting the expression of ATG4D in HPAC and SW1990 cells. We found that the mRNA and protein expression levels of CD44 and CD133 decreased after downregulating ATG4D by siRNA in HPAC and SW1990 cells (Figures 7B,C). Sphere formation assay is a key method to reveal the self-renew and differentiation ability of PaCSCs. Thus, we measured self-renewal capacity and observed significantly increased sphere-forming capacity for HPAC cells treated with T0070907 compared to Rosiglitazone and negative control cells (Figure 7D).

Additionally, we detected the cell viability after treating HPAC or SW1990 cells with Gemcitabine combined with Rosiglitazone or not. The results indicated that combination of Rosiglitazone and Gemcitabine in HPAC and SW1990 cells significantly inhibited tumor cell viability compared to Gemcitabine alone (Figure 7E). These results gave us some hints that the pro-proliferation role of PPARγ in PDAC might improve the chemotherapy sensitivity of PDAC *via* inhibiting mitophagy-regulated cancer stemness.

## Discussion

PPARγ has been implicated in the carcinogenesis and progression of various solid tumors. Emerging evidence has shown that PPARγ plays an oncogenic role *via* mitochondrial anti-oxidative function (Cao et al., 2018; Zou et al., 2019). The inhibition of mitochondrial anti-oxidative function produces ROS, damaging the electron transport chain. Then, decreased MMP leads to the collapse of mitochondrial structure and function to induce cell apoptosis (Abdel Hadi et al., 2021). The mitochondrial anti-oxidative function of PPARγ on PDAC progression remains unclear. Our study found that high expression of nuclear PPARγ in pancreatic cancer tissues was correlated positively with tumor size and predicted poor prognosis in patients. *In vitro* and *in vivo* studies further confirmed that PPARγ activation could promote cell proliferation *via* stabilizing the MMP and inducing the mitochondrial redox capability to inhibit mitochondrial ROS-dependent cell apoptosis. SOD2, the primary mitochondrial oxidative scavenger, over-expresses in a variety of tumors including PDAC. It could influence the malignant behaviors of tumors *via* exerting mitochondrial anti-oxidative function (Hart et al., 2015; Li et al., 2015). In our study, SOD2 expression could be directly regulated by the transcription factor PPARγ, inhibiting mitochondrial ROS-dependent cell apoptosis. These results indicated that PPARγ/SOD2 pathway could protect against mitochondrial ROS-dependent apoptosis to promote the proliferation of pancreatic cancer cells.

Mitophagy plays an important role in the quality and function of mitochondria, influencing tumor cell metabolism, oxidative stress and biosynthesis process (Kubli and Gustafsson, 2012). mTOR pathway, the metabolism-related classical pathway, participating in autophagic process, regulates termination of autophagy and reformation of lysosomes (Yu et al., 2010). ULK1 modulates the initiation of autophagy by recruiting autophagy-related genes (ATGs), and the phosphorylation of ULK1 would be activated by mTOR blockade, thereby inducing the autophagy occurrence (Kim et al., 2011). Consistently, our study suggested that the inhibition of PPARγ, followed by SOD2 blockade, could activate mitophagy process *via* the mTOR/ULK1 signaling pathway. Notably, autophagic degradation and the removal of damaged mitochondria might aid cellular response to mitochondrial oxidative stress (Ashrafi and Schwarz, 2013). Mitochondrial ROS under oxidative stress could be regulated by mitophagy contributing to cell apoptosis (Chen et al., 2012; Zhou et al., 2019). The molecular mechanism between mitophagy regulated by PPARγ/SOD2 and apoptosis remains unknown. ATG4D, one of the ATG4 family members, plays an important regulatory role during the formation of autophagosome. The sequence of ATG4D contains mitochondrial targeting sequences (MTS) and is located on the downstream of the caspase cleavage site. The ATG4D fragment (ΔN63 Atg4D) could be cleaved by the caspase, then translocated to the damaged mitochondrial (Betin et al., 2012). The fragment expresses BH3 domain, inducing interaction with BCL-2 family members to induce apoptosis (Betin and Lane, 2009). In our study, we found that PPARγ/SOD2 pathway activation could downregulate the expression of ATG4D. Furthermore, after the blockade of PPARγ/SOD2 pathway, ATG4D was located in mitochondria to activate the process of mitophagy, contributing to the increase of mitochondrial ROS production, damage of MMP stabilization and promotion of cancer cell apoptosis. Therefore, we conclude that PPARγ/SOD2 could protect against mitochondrial ROS-dependent apoptosis *via* inhibiting ATG4D-mediated mitophagy to promote PDAC proliferation.

Notably, mitophagy could be used by PaCSCs for particular adaptation of metabolic stress, as a major limitation of anti-cancer treatments, contributing to chemotherapy resistance (Ferro et al., 2020). PaCSCs present more intracellular active mitophagic flux than non-stem cancer cells (Valle et al., 2020). Previous studies found that PPARγ activation could induce the differentiation of cancer stem cells to mature cancer cells, promoting the proliferation capability of breast cancer (Moon et al., 2014; Papi et al., 2014). Rosiglitazone combined with gemcitabine reduces immune suppression and modulates T cell function in pancreatic cancer (Bunt et al., 2013). In our study, we found that PPARγ might diminish the PDAC stemness *via* decreasing the expression level of CD44 and CD133 in pancreatic cancer cells, enhancing the killing effect of Gemcitabine on pancreatic cancer cells. The weakened ATG4D-mediated mitophagy dominated by PPARγ might play a role in the process of inhibiting pancreatic cancer cell stemness. Thus, PPARγ agonists combined with mitophagy inhibitors probably promote PDAC proliferation to improve chemosensitivity, defining a novel therapeutic target of PDAC. In our future study, we will explore the mechanisms of specific interaction between PPARγ, mitophagy and pancreatic cancer stemness.

## Data Availability

The original contributions presented in the study are included in the article/Supplementary Material, further inquiries can be directed to the corresponding authors.
