# Supplementary figures and images for: PPARγ/SOD2 Protects Against Mitochondrial ROS-Dependent Apoptosis via Inhibiting ATG4D-Mediated Mitophagy to Promote Pancreatic Cancer Proliferation
Source: Front Cell Dev Biol. 2022 Feb 2;9:745554. doi: 10.3389/fcell.2021.745554 (PMC8847684; doi:10.3389/fcell.2021.745554)

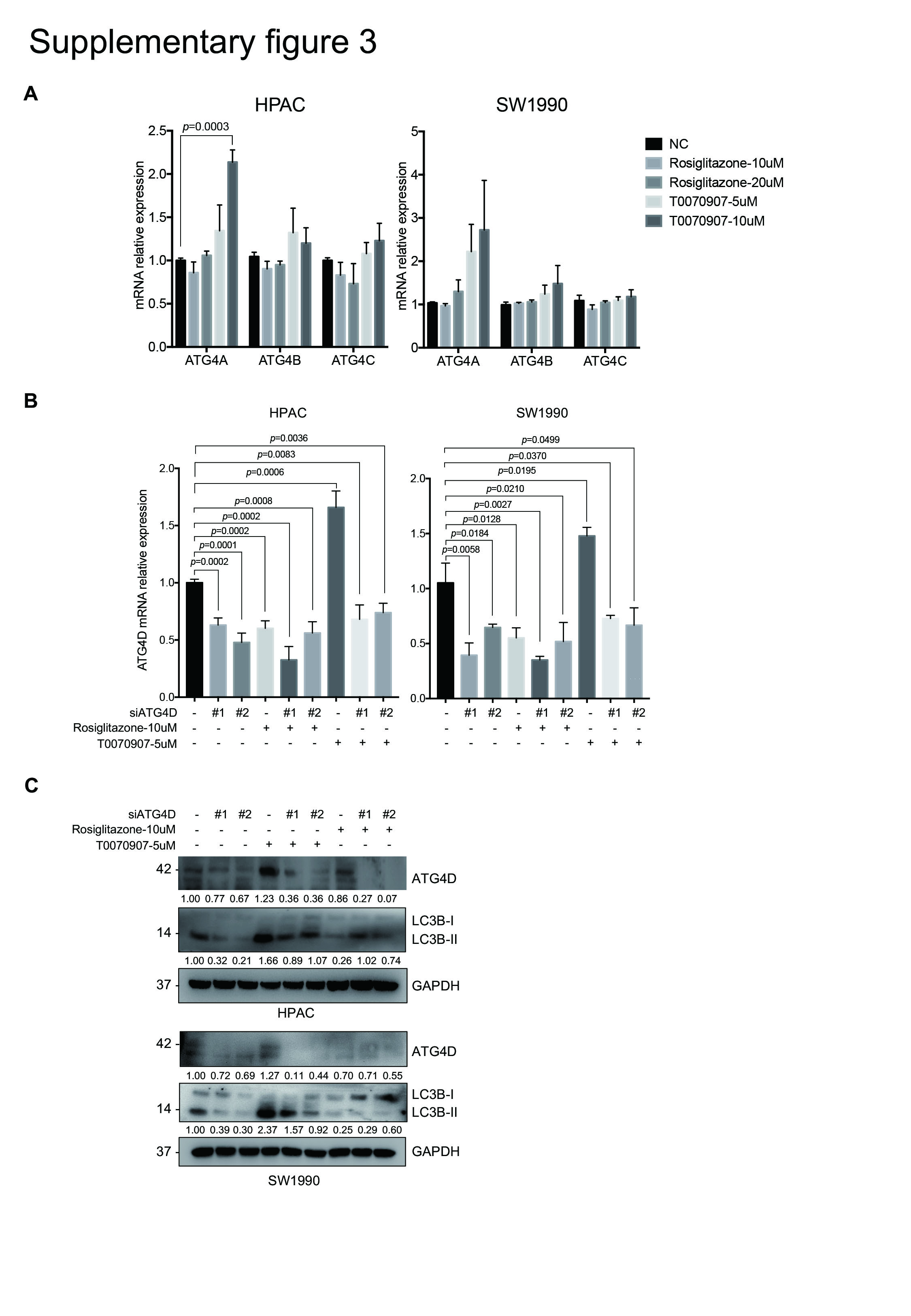

Supplement: Supplementary file 1 [file Image3.jpeg]

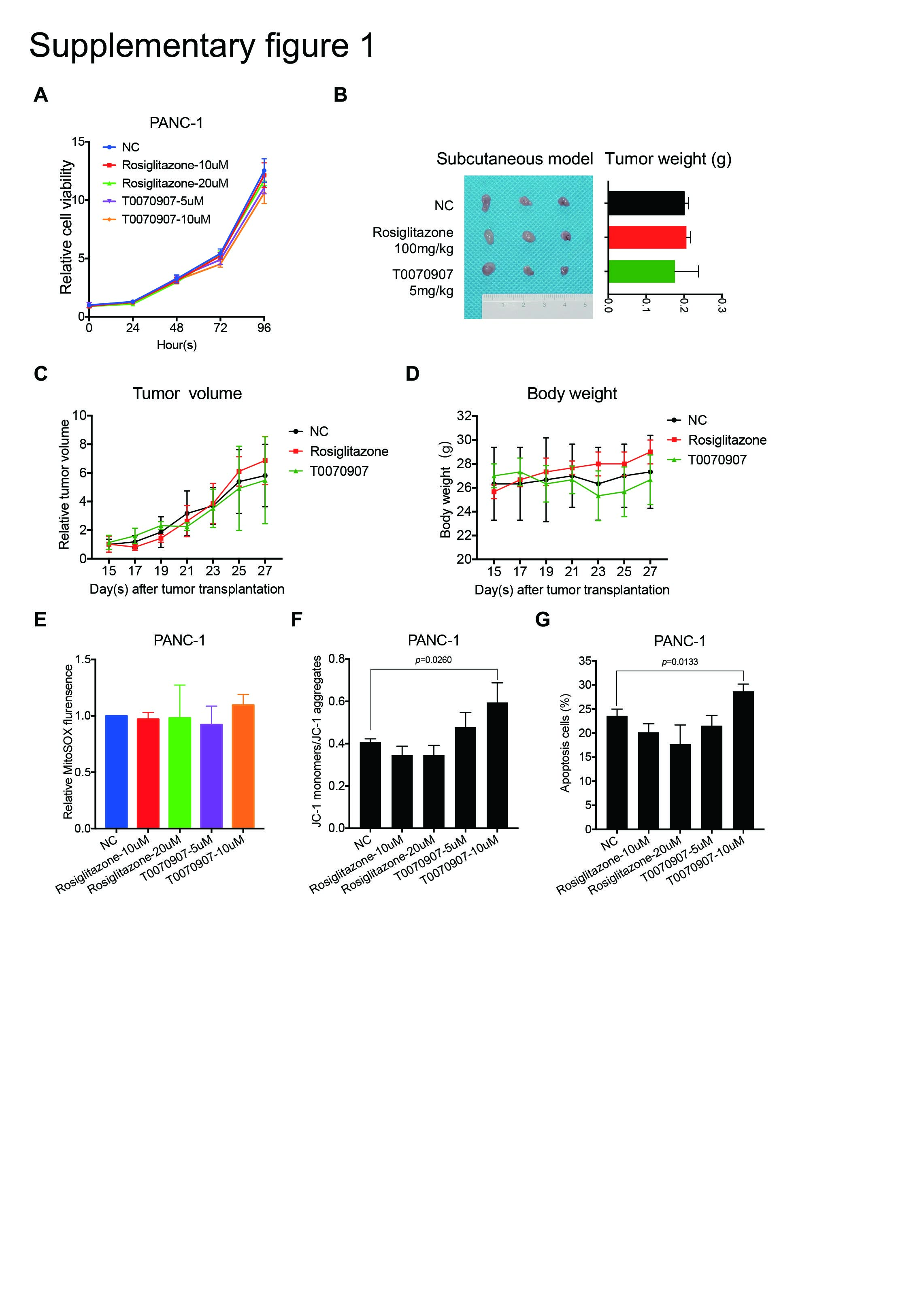

Supplement: Supplementary file 2 [file Image1.jpeg]

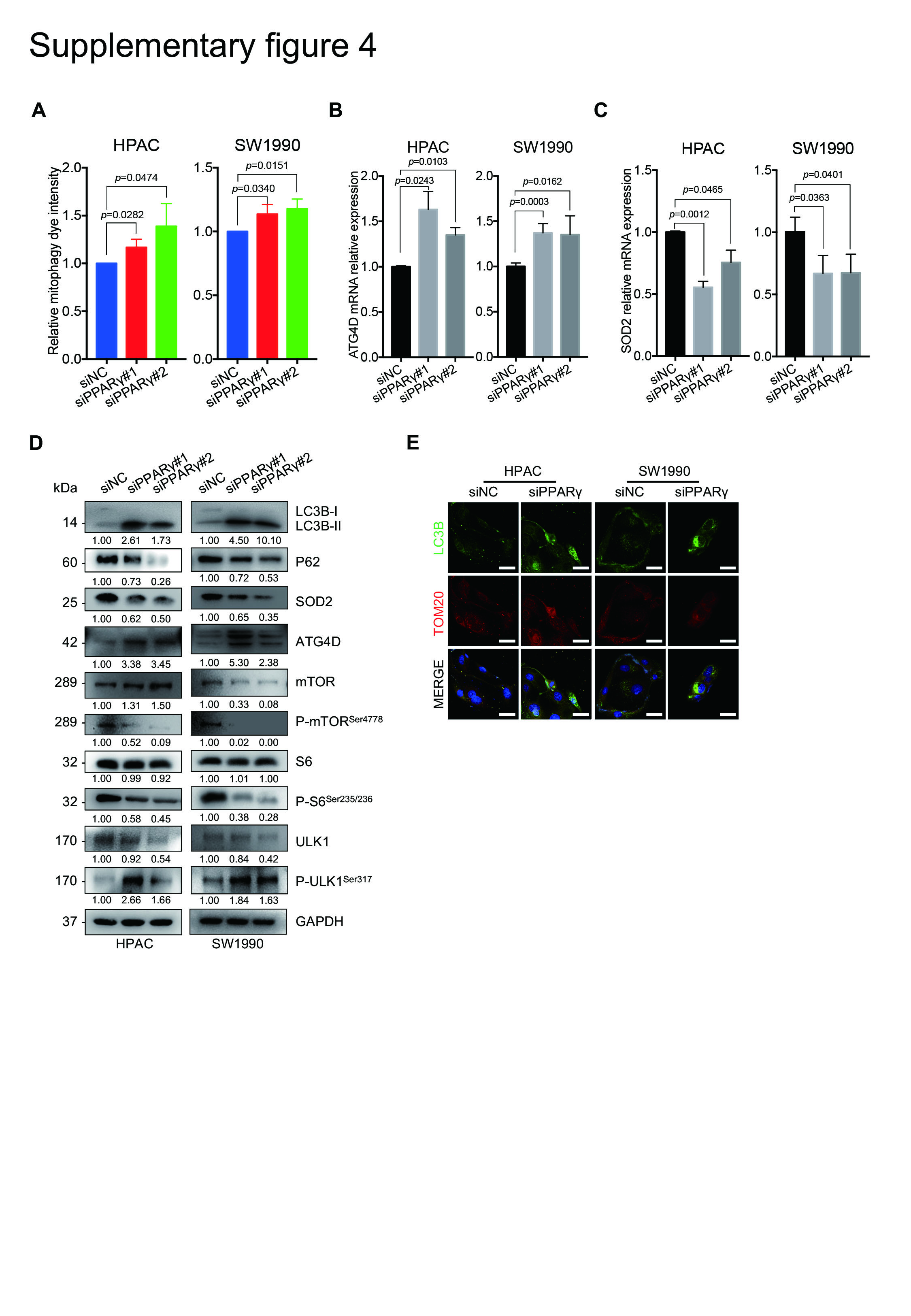

Supplement: Supplementary file 3 [file Image4.jpeg]

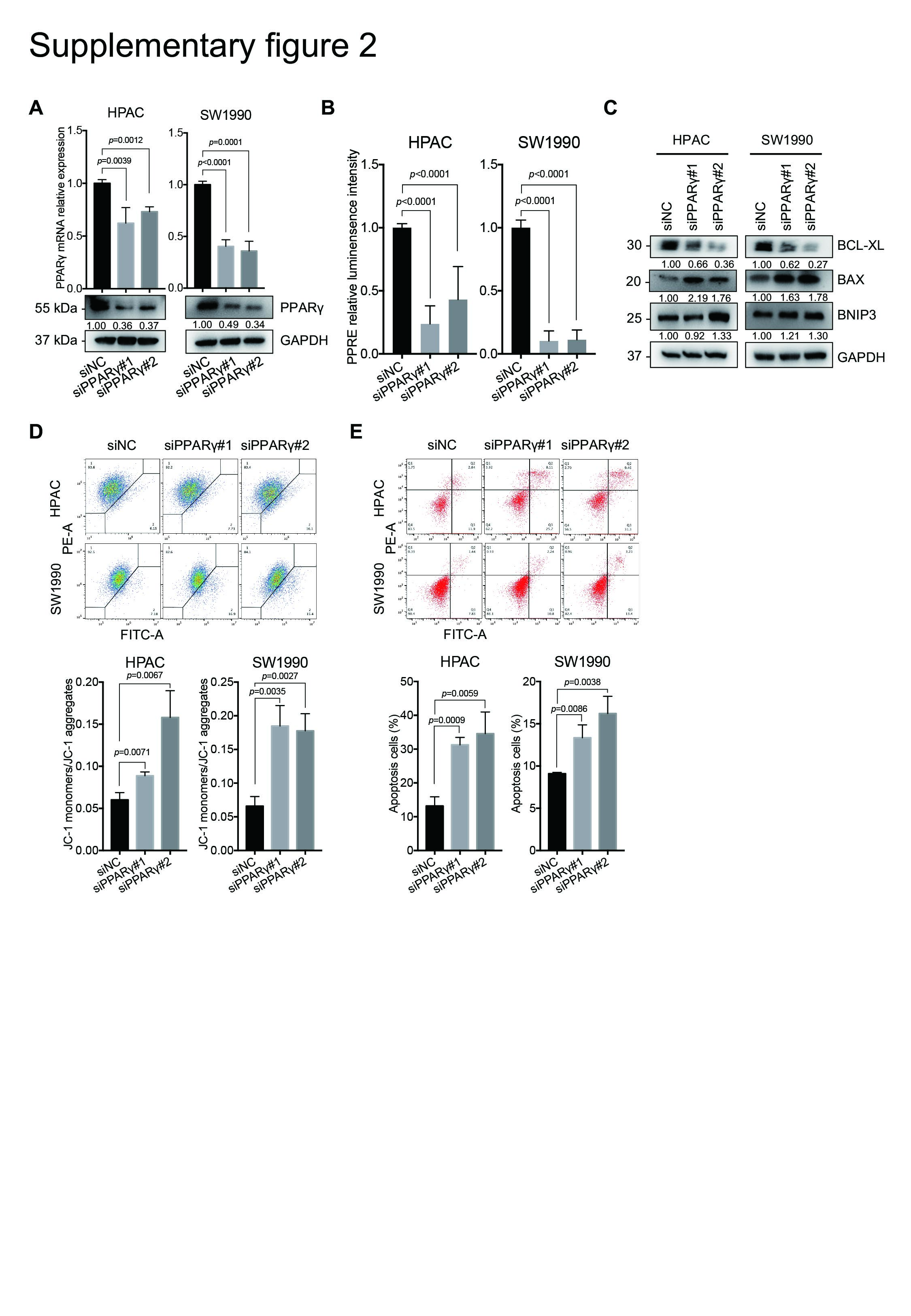

Supplement: Supplementary file 4 [file Image2.jpeg]
